# Finance and health dialogue needed to build resilient health systems 

**DOI:** 10.2471/BLT.24.291539

**Published:** 2024-04-17

**Authors:** Chris James, Francesca Colombo, David Morgan, Camila Vammalle

**Affiliations:** a Directorate for Employment, Labour and Social Affairs, Organisation for Economic Co-operation and Development (OECD), 2 rue André Pascal, 75016 Paris, France.; b Public Governance Public Management and Budgeting Division, OECD, Paris, France.

The coronavirus disease 2019 (COVID-19) pandemic has demonstrated that high-performing, resilient health systems are essential to the health of populations as well as to resilient economies and societies.^1^ Yet raising sufficient public funds for health and sustaining such spending over time is challenging: health systems compete for government resources, and countries with limited fiscal space will particularly struggle to do so. In the current economic context of higher and less predictable inflation, coupled with an uncertain economic outlook, government budgets are adversely affected, with knock-on effects to the health sector. These effects are felt both in countries of the Organisation for Economic Co-operation and Development (OECD), which have largely achieved universal health coverage (UHC), and in other countries still on the path to providing high quality, accessible health care for all their citizens.^2^

Financing of health systems in a way that protects citizens and improves the resilience of health systems to unexpected shocks must therefore remain a central policy objective. Doing so requires a clear identification of the options policy-makers have at their disposal, including dialogue with finance ministries.

The pressing policy question is how to find a fiscally sustainable path to fund the increases in health spending that are needed to secure equitable health outcomes. To help countries finance more resilient health systems, the OECD developed four policy levers for governments: (i) increase government spending and allocate part of these additional funds to health; (ii) increase the allocation to health within existing government budgets; (iii) reassess the boundaries between public and private spending; and (iv) find efficiency gains and make efforts to cut ineffective spending in health systems.^2^ Further details about the policy levers are presented in Box 1. These four levers are not mutually exclusive, depending on country circumstances, some are more compelling than others. Each lever has its own risks and constraints. Ultimately, the choices of policy-makers will be influenced by the political will and the economic feasibility for change, especially in the short to medium term.

Box 1 **OECD-recommended policy levers for increasing spending for resilient health systems**

***Increase government spending and allocate part of these additional funds to health***

Doing so requires the mobilization of government revenues through greater tax revenues or additional debt financing which in turn requires collaborative efforts to support fiscal reforms in countries. This option might be more compelling for low- and middle-income countries with typically a low tax-to-GDP ratio and high reliance on out-of-pocket spending for health, than for many high-income countries that already have high levels of taxation and a low appetite for further increasing borrowing or taxes in a cost-of-living crisis.

***Increase the allocation to health within existing government budgets***

Doing so implies reallocating resources from other sectors. However, across OECD countries, health already represents 15% of government budgets and health is competing with major new spending priorities, such as funding a green transformation, and, for some countries, increasing defence spending given current global events. In contrast, health authorities in countries with low budget allocations to health could push for increased budget shares to help reduce out-of-pocket private spending, and increase their ability to make the economic case for such spending with finance authorities. Many low-income countries where health spending accounts on average for only around 6% of government budgets, could do so.

***Reassess the boundaries between public and private spending***

For countries with already high levels of public spending on health, without additional public resources for health more health-care spending will by default shift to the private sector. Unless carefully managed, this situation risks endangering access to care in countries that have achieved UHC. For low- and middle-income countries that already have a high reliance on out-of-pocket spending, any shifts to private funding risks increasing financial hardship and reducing progress to the sustainable development goals. Nevertheless, a debate on longer-term directions on the public-private boundary needs to be had in all countries, particularly in terms of what are the best buys for limited public budgets.

***Find efficiency gains and make efforts to cut ineffective spending in health systems***

While policy-makers in countries that spend relatively little on health may think this is only an issue for high-income countries, in fact, focusing spending on where returns on health are the highest from the very start in the path towards UHC is essential to both achieve and sustain UHC. A first step to achieve efficiency gains involves cutting wasteful spending, notably by reducing medical errors, cutting the inappropriate use of antimicrobials and limiting unwarranted variation in medical practices.^3^ At the same time, proven approaches to increase productivity include policies on health workforce, pharmaceuticals and new technologies. For example, laws and regulations that extend the scope of practice for non-physicians (such as nurses and pharmacists) can produce cost savings with no adverse effects on quality of care. For pharmaceuticals, price, market entry and prescribing regulations have all helped increase penetration of generics in the market, thereby saving costs. Digitalization can support new care delivery models, notably in the form of telemedicine and robotic tools for some limited procedures, with better use of health data improving the management of critical care resources, while also reaping the benefits of artificial intelligence. Many of these policies are ambitious and require transformative policy changes, but they are key to maximizing value for money in health systems.

GDP: gross domestic product; OECD: Organisation for Economic Co-operation and Development; UHC: universal health coverage.Note: Adapted from OECD, 2024.^2^

The options are not easy to implement. They require a conversation about how much society is willing to pay for health. They imply sometimes facing and addressing difficult prioritization decisions both within and beyond the health sector. The political and technical feasibility of each policy lever cannot be resolved without a much more robust dialogue and partnership between the government agency responsible for the overall government budget at the national level and the government agency or agencies that are primarily responsible for the health system. This dialogue is the way to find common solutions that combine raising additional funds with efforts to free up current resources by reducing ineffective spending and focusing investment where the returns are the highest.

To aid governments to implement these policy levers, the OECD created the Joint Network of Senior Budget and Health Officials.^4^ This network brings together policy-makers from both the finance and health sectors to discuss health financing challenges and seek policy solutions. Established in 2011, the network provides an open space for government officials from ministries of finance and health as well as social security agencies to discuss health financing policies. The network combines peer-to-peer learning with the latest analytical work of the OECD. More recently, the Asia-Pacific Network; the Latin America and Caribbean Network; and Central, Eastern and South-Eastern Europe Network have been established in close collaboration with international partners including the World Health Organization (WHO), the World Bank, regional banks, and the Global Fund to Fight AIDS, Tuberculosis and Malaria.

Alongside these policy dialogues, the OECD network provides cross-country analysis and tailored support to countries on specific health financing issues, notably on health budgeting and the fiscal sustainability of health systems. A recent example has been the establishment of an agreed set of high-level good budgeting practices in the health sector, based on close consultation with country participants of the network.^5^ These good practices are designed to help policy-makers assess their own budgeting arrangements for health – the natural entry point for reform discussions between finance and health policy-makers – with good practices detailed for each stage of the budget cycle. 

The network also provides tailored support to countries. In France, for example, the High Council for Health Insurance recommended reforming how the country calculates and implements its National Objective for Health Spending.^5^ The network supported the high council in reform design by providing international benchmarks and organizing international experts’ workshops.

Recent major reforms in New Zealand offer another practical example of finance and health authorities working together to instigate change. In 2022, New Zealand began a series of major health system reforms. As well as organizational restructuring, these reforms targeted the budget process for health, with budget setting moving away from baselines where there were no automatic adjustments for major cost drivers, to costing based on a model developed by the treasury and the health ministry.^6^ Leading up to this reform, finance and health officials from New Zealand engaged bilaterally with the OECD and in small country group workshops to discuss these budgeting issues.

Enhanced dialogue with finance authorities is key for addressing any of the four policy levers described above. Good budgeting practices are also particularly important to maximize the efficiency of current health spending, and enable more ambitious policy changes in the medium to longer term that support higher health outcomes for each United States dollar spent. Good budgeting practices across the budget cycle facilitate better decisions on whether, when and by how much public funding for health can increase, as well as identifying where priorities can be shifted.

The required reforms to health systems are not always straightforward to implement. Finance authorities need to set out clearly the fiscal constraints within which they are operating. Health authorities need to demonstrate the wider benefits of good health and health care being a driver of economic growth, and resilient health systems being critical to protecting society in the face of future shocks, alongside the intrinsic value of better health. To this end, the recent report from the WHO Council on the Economics of Health for All^7^ provides practical recommendations on how to value health for all. Health authorities also need to assure finance authorities that any additional funds will be spent effectively, and at the same time demonstrate their commitment to take bold action to cut wasteful spending.

The rewards of a more effective dialogue are substantial: strengthening health system resilience protects economies from destabilizing health shocks, and protects people from ill health and premature death. Health and finance authorities alike must be aware of the risks of governments not spending enough but also of not spending well on health, with the knock-on effects extending well beyond the health sector.
